# Differential Effects of Brain Disorders on Structural and Functional Connectivity

**DOI:** 10.3389/fnins.2016.00605

**Published:** 2017-01-09

**Authors:** Sandro Vega-Pons, Emanuele Olivetti, Paolo Avesani, Luca Dodero, Alessandro Gozzi, Angelo Bifone

**Affiliations:** ^1^NeuroInformatics Laboratory, Fondazione Bruno KesslerTrento, Italy; ^2^Centro Interdipartimentale Mente e Cervello, Università di TrentoTrento, Italy; ^3^Pattern Analysis and Computer Vision, Istituto Italiano di TecnologiaGenova, Italy; ^4^Istituto Italiano di Tecnologia, Center for Neuroscience and Cognitive Systems @UniTnRovereto, Italy

**Keywords:** structural connectivity, functional connectivity, kernel two-sample test, graph kernel, test statistic, agenesis of the corpus callosum

## Abstract

Different measures of brain connectivity can be defined based on neuroimaging read-outs, including structural and functional connectivity. Neurological and psychiatric conditions are often associated with abnormal connectivity, but comparing the effects of the disease on different types of connectivity remains a challenge. In this paper, we address the problem of quantifying the relative effects of brain disease on structural and functional connectivity at a group level. Within the framework of a graph representation of connectivity, we introduce a kernel two-sample test as an effective method to assess the difference between the patients and control group. Moreover, we propose a common representation space for structural and functional connectivity networks, and a novel test statistics to quantitatively assess differential effects of the disease on different types of connectivity. We apply this approach to a dataset from BTBR mice, a murine model of Agenesis of the Corpus Callosum (ACC), a congenital disorder characterized by the absence of the main bundle of fibers connecting the two hemispheres. We used normo-callosal mice (B6) as a comparator. The application of the proposed methods to this data-set shows that the two types of connectivity can be successfully used to discriminate between BTBR and B6, meaning that both types of connectivity are affected by ACC. However, our novel test statistics shows that structural connectivity is significantly more affected than functional connectivity, consistent with the idea that functional connectivity has a robust topology that can tolerate substantial alterations in its structural connectivity substrate.

## 1. Introduction

Neuroimaging methods, like Magnetic Resonance Imaging (MRI), provide a powerful tool to investigate brain connectivity. They have been widely applied to study the mutual relationship between structural and functional connections of brain regions in healthy subjects and patients, as well as in animal models. In this context, structural connectivity is defined by the physical connection of remote brain regions by white matter fibers, as measured by diffusion weighted MRI (Assaf and Pasternak, 2008). Conversely, functional connectivity is inferred from correlations between fMRI signals, typically under resting state conditions, and is thought to associate brain areas that share common functional properties (van den Heuvel and Hulshoff Pol, 2010).

Alterations in functional and structural connectivity have been observed in neurological and psychiatric disorders (Fornito and Bullmore, 2012; Tost et al., 2012), and in conditions where maladaptive changes in the brain have occurred following traumatic neural injuries (Seifert and Maihöfner, 2011) or long-term exposure to substances of abuse (Sutherland et al., 2012). Abnormalities in the brain connectivity represent a promising putative endophenotype for certain mental disorders, like autism (Rane et al., 2015) and schizophrenia (Fornito et al., 2012), that are not associated with focal neuropathological features, but are rather thought to be developmental disorders characterized by pathological patterns of neural connectivity. The mutual relation between functional and structural connectivity, and the effects of brain disease on these different forms of connectivity remain the subject of investigation.

Intuitively, functional connectivity should correlate with structural connectivity patterns. Indeed, several studies have demonstrated that structurally connected cortical regions exhibit stronger and more consistent functional connectivity than structurally unconnected regions (Koch et al., 2002; Honey et al., 2007). However, robust functional connectivity can also be found between regions not linked by cortico-cortical projections, and the relation between the two kinds of connectivity is not strictly biunivocal.

In the adult, healthy brain, structural, and functional connectivity appear to be positively correlated, at least at an aggregate level. This is consistent with the idea that brain regions that are strongly structurally connected should tend to exhibit stronger patterns of functional connectivity (Honey et al., 2009).

The picture that emerges from studies in patients affected by brain disease and neurological conditions is more complex. A striking example is that of subjects with Agenesis of the Corpus Callosum (ACC), a congenital condition whereby the main bundle of white matter fibers connecting the two cerebral hemispheres does not form during brain development. This condition is accompanied by a reorganization of white matter architecture, with the presence of anomalous longitudinal bundles of fibers known as Probst bundles. Early resting-state functional MRI investigations in ACC subjects, whose structural connectivity is drastically impaired, have detected alterations in inter-hemispheric connectivity (Quigley et al., 2003). However, more recent studies have surprisingly shown intact bi-lateral functional connectivity patterns (Uddin et al., 2008; Tyszka et al., 2011; Owen et al., 2013). This evidence suggests the hypothesis that small, preserved commissural fibers may suffice to support normal levels of inter-hemispheric connectivity, or that multisynaptic connections may be able to maintain a high degree of bilateral coherence, even in the absence of direct cortico-cortical structural links.

Altogether, these results challenge the view that structural and functional connectivity are straightforwardly related. Therefore, the ability to quantitatively compare differences in structural and functional connectivity would contribute to the understanding of the mismatch observed in patients. Moreover, it would be important for the study of the plastic mechanisms underlying brain development and the recovery of functional connectivity in the case of congenital or acquired loss of white matter tracts.

In this paper, we propose a novel approach to study the mutual relationship between structural and functional connectivity in a group of subjects affected by a brain condition compared to their healthy controls. The main aim is to provide a support to a quantitative analysis of the differences on brain connectivity. The localization of connectivity differences is out of the scope of this work.

By way of example, we apply this approach to functional and structural connectivity data from BTBR T+Itpr3tf/J mice (BTBR) (Han et al., 2014; Squillace et al., 2014), an inbred mouse line with ACC, using the normo-callosal C57Bl6/J (B6) mice as comparators. This is an ideal model to validate our approach, and to investigate the differential effects of a pathological condition on structural and functional connectivity. Indeed, the small genetic variability in the BTBR inbred line results in a very consistent phenotype that lends itself to a quantitative analysis in controlled experimental groups.

The specific question we address is to what extent the aberrant structural connectivity of the acallosal BTBR model is paralleled by a similar disruption and reorganization of functional connectivity. Previous studies (Dodero et al., 2013) have shown that BTBR mice exhibit a complete lack of the corpus callosum and a severely reduced hippocampal commissure, with a strong reduction of the white matter bundles connecting the two hemispheres. Conversely, functional connectivity shows a good degree of homotopy, with normal interhemispheric connectivity in the posterior cortices and a reduction in the strength of interhemispheric connectivity in the frontal part of the brain (Sforazzini et al., 2016). Qualitatively, functional connectivity appears to be relatively robust to disruption of the underlying structural connectivity, but a statistically sound method to compare the two is missing.

In principle, given structural and functional connectivity data from the disease (BTBR) and the control group (B6), this problem can be tackled in 2 steps:

1. *Class discrimination with single modality:* Discrimination between BTBR and B6 by independently using the structural and functional connectivity. The more affected the connectivity is in the BTBR class the more separable the classes should be.
2. *Modality comparison:* Quantitative comparison of how discriminative the two connectivity modalities are for the BTBR vs. B6 problem. Intuitively, if the functional connectivity in BTBR is as altered as the structural connectivity, the two classes (BTBR and B6) should be equally separable when using any of the two types of connectivity. Conversely, if the functional connectivity in BTBR is less affected, it should be easier to discriminate between BTBR and B6, by using the structural connectivity.

The class discrimination problem based on a single connectivity modality has been previously addressed by using different approaches. For example, seed-region methods, statistical tests on graph indexes and machine learning classifiers have been proposed (see review in Section 3.1). In this paper, we introduce the kernel two-sample test (KTST) (Gretton et al., 2012) as a method to tackle the class discrimination problem. Thanks to the definition of a characteristic graph kernel, the KTST can be directly applied on the brain connectivity to determine whether the data from the disease class (BTBR) and control (B6) are drawn from the same probability distribution or not. We argue that this approach can be more appropriate than existing methods in the case of small datasets.

Ideally, once we obtain the results from the class discrimination problem on each one of the two connectivity modalities, the comparison of such results should tell which type of connectivity is more affected. However, class discrimination results are not always straightforwardly comparable. Indeed, when the results of the two modalities are close to extreme values, i.e., chance level or perfect discrimination, there is no reliable method to quantitatively compare them. One of the main hurdles that hampers the direct comparison of results obtained with both modalities is the difference in the nature of the two types of connectivity. In order to address this problem, we propose a novel common representation space for structural and functional connectivity networks. In this common space, we introduce a new test statistic derived from KTST, that directly addresses the problem of comparison between the modalities. A quantitative assessment of the comparison provides the statistical evidence that the difference in functional connectivity is much smaller that the difference in structural connectivity. Upon this result we may claim that patterns of functional connectivity are relatively robust with respect to disruption of the underlying white matter connectivity, as homotopy is largely preserved in spite of the lack of CC. Our findings are also consistent with the observation of intact resting state networks in cases of ACC, and of post-operative recovery of functional connectivity organization following surgical rescission of the CC.

The rest of the paper is organized as follows. In Section 2, we describe the dataset that we use in our analysis, giving details about the acquisition, preprocessing, and computation of connectivity networks. In Section 3, we introduce the KTST, together with a characteristic graph kernel, as an alternative approach to the class discrimination problem. Moreover, we introduce our solution to the modality comparison problem, based on the definition of a common representation space for structural and functional connectivity and a novel test statistic. In Section 4, we provide the results of the application of our proposals to the dataset previously described. In Section 5, we discuss these results and their implications. Finally, in Section 6, we conclude this work and mention future perspectives.

## 2. Materials

The MR datasets used to construct the adjacency matrices representing the structural and functional connectivity networks hereby investigated have been reported in Dodero et al. (2013) and Sforazzini et al. (2016), where protocols and acquisition methods are described in detail. All *in vivo* studies were carried out in accordance with the European directive 86/609/EEC governing animal welfare and protection, which is acknowledged by the Italian Legislative Decree no. 116, 27 January 1992. Animal research protocols were also reviewed and consented to by a local animal care committee. All surgical procedures were performed under anesthesia.

In short, all MRI data were acquired at 7T with a Pharmascan Bruker system equipped with four RF channels, a 72 mm birdcage transmit coil, and a custom- built saddle-shaped solenoid coil for signal reception.

Structural connectivity was derived from Diffusion Tensor Imaging data from paraformaldehyde (4% PFA) fixed brains to avoid any physiological or motion artifacts. Experimental and control groups consisted of eight adult male BTBR and eight B6 mice. Diffusion tensor images (DTI) were acquired with 81 different gradient orientations at a *b*-value of 1262 s/mm^2^ (*h* = 5 ms, *D* = 10 ms), in-plane spatial resolution of 130 × 130 μm^2^, and slice thickness of 350 μm using using a four-shot EPI sequence with *TR* = 5500 ms and *TE* = 26 ms, 20 averages. Anatomical reference images were acquired with 3D RARE spin-echo sequence, *TR* = 550 ms, *TE* = 33 ms, RARE factor = 8, echo spacing 11 ms, and voxel size of 90 μm^3^ (isotropic). Images were co-registered to a mouse brain template (Sforazzini et al., 2016).

Diffusion Tensor Tractography (DTT) of BTBR and B6 control subjects was performed by estimating axonal fibers projections with a deterministic fiber assignment using the continuous tracking algorithm (Mori et al., 1999). Criteria for terminating the tracking of individual fibers included an anisotropy threshold (values below 0.15) and a maximum stiffness condition, so that the tracking was terminated when the diffusion directions in consecutive steps differed by more than 35 μm. Fibers shorter than 3 mm were filtered out leading to a set of about 80000 streamlines.

Resting State fMRI time series were acquired on male 26-week old B6 and BTBR mice (*n* = 10 each group), which were anesthetized with isoflurane (5%), intubated and artificially ventilated. At the end of animal preparation, isoflurane was discontinued and substituted with halothane (0.7%) ca. 100 min prior to the beginning of rs fMRI data acquisition.

Co-centered single-shot BOLD time-series were acquired using an echo planar imaging (EPI) sequence with the following parameters: *TR/TE* 1000/15 ms, flip angle 60°, matrix 100 × 87, field of view 2.3 × 2 cm^2^, 16 coronal slices, slice thickness 0.75 mm, 360 volumes, and a total rsfMRI acquisition time of 6 min. Image pre-processing was performed as described previously (Sforazzini et al., 2014). Briefly, anatomical brain images were co-registered to the same mouse brain template of the DTI data using FSL. The generated warp fields were applied to the co-centered rsfMRI time series. After co-registration, all the functional images were motion-corrected and the estimated movement parameters, together with mean ventricular signal, were considered as nuisance signals and regressed out. The image time series were then band-pass filtered to a frequency window of 0.01–0.08 Hz and spatially smoothed using a Gaussian kernel of full-width at half maximum of 0.6 mm.

Finally, brain connectivity networks were built in the following way. Fifty anatomical volumes of interest (VOIs) were defined using bilateral brain regions from the mouse atlas (Dorr et al., 2008). Each of these VOIs represents a node in the graph representation of structural and functional connectivity data. Adjacency matrices for the structural connectivity graphs were constructed by calculating the number of streamlines connecting each pair of nodes in every subject. For the functional connectivity graphs, mean time courses from each VOI were calculated and variance-normalized. Pairwise Pearson correlation coefficients were then calculated to generate VOI—VOI correlation matrices for each subject.

## 3. Methods

As presented in the previous section, the dataset analyzed in this paper is composed by two different types of connectivity data (structural and functional) from subjects belonging to two categories (or classes), i.e., BTBR and B6. This dataset has 16 graphs representing the structural connectivity, with 8 graphs for each class and 20 graphs of functional connectivity, with 10 graphs belonging to each class. The methods we propose in this paper have been mainly developed to study this specific dataset. However, in this section, we describe them using a more general notation, because they are not restricted to this particular dataset, but can be directly applied to similar case studies.

Both structural and functional connectivity data can be defined by using concepts from graph theory. In both cases, the connectivity data is represented by simple, undirected, node-labeled and edge-weighted graphs *G* = (*V, E*, ℓ, ω),

where *V* is the set of nodes, *E* ⊂ *V* × *V* is the set of edges, and ℓ and ω are node and edge labeling functions, respectively. In the case of the BTBR vs. B6 dataset, node labels are identifiers to the 50 volumes of interest (VOI) defined in the network construction. On the other hand, edge labels are real values that represent the statistical dependency between every pair of brain regions. The nature of the edge labeling function ω is in fact, the main difference between structural and functional connectivity.

With this kind of graphs, it is possible to define a mapping between nodes representing the same brain region in different graphs. This is known in the literature as *fixed-cardinality vertex sequence* (FCVS) (Richiardi et al., 2013) property. It means that all graphs have the same number of nodes and there is a one-to-one correspondence between nodes across the graphs. This correspondence is given by the node labeling function ℓ. If two nodes from different graphs have the same label, it means that the two nodes represent the same brain region. Considering the FCVS property and assuming an ordering on the node labels, each graph *G* can be well-characterized by its adjacency matrix *A*, where each cell *A*_*uv*_ contains the weight ω(*e*) associated to the edge *e* = (*u, v*) that connects the nodes with the *u*-th and *v*-th labels.

In general, we assume that we have a set of such graphs representing the structural connectivity data 𝔾^*s*^ = $\left\{G_{1}^{s},G_{2}^{s},\ldots ,G_{n}^{s}\right\}$ with the associated class labels $Y^{s}=\left\{y_{1}^{s},y_{2}^{s},\ldots ,y_{n}^{s}\right\}$, where $y_{i}^{s}$ is the class (BTBR or B6) of $G_{i}^{s}=\left(V_{i}^{s},E_{i}^{s},\ell_{i}^{s},\omega_{i}^{s}\right)$ for all *i* = 1, …, *n*. Moreover, we also have a set of functional connectivity graphs 𝔾^*f*^ = $\left\{G_{1}^{f},G_{2}^{f},\ldots ,G_{m}^{f}\right\}$ and the corresponding class labels $Y^{f}=\left\{y_{1}^{f},y_{2}^{f},\ldots ,y_{m}^{f}\right\}$ where also $y_{j}^{f}$ is the class label (BTBR or B6) of graph $G_{j}^{f}=\left(V_{j}^{f},E_{j}^{f},\ell_{j}^{f},\omega_{j}^{f}\right)$ for all *j* = 1, …, *m*. Therefore, we have two binary-class datasets, D_S_ = {𝔾^*s*^, *Y^s^*} containing the structural connectivity data and D_F_ = {𝔾^*f*^, *Y^f^*} containing the functional connectivity data, respectively. Despite some pairs of structural and functional connectivity graphs can belong to the same subject, we do not use this information in our methods because there could be subjects contributing with only one modality. This is exactly the case of the particular dataset we are studying in this paper, where there are *n* = 16 graphs of structural connectivity and *m* = 20 graphs of functional connectivity.

As described in Section 1, we are interested in the *class discrimination* and the *modality comparison* problems. Therefore, in Section 3.1, we briefly review the most prominent techniques proposed in the literature for the class discrimination problem based on brain connectivity and introduce the necessary concepts for the next sections. In Section 3.2, we introduce our alternative approach to this problem based on the use of the Kernel Two-Sample Test (KTST) and discuss its main characteristics. In Section 3.3, we introduce our solution to the modality discrimination problem. This is based on a common representation space for structural and functional connectivity and a new test statistic that allows the direct comparison of the two modalities.

### 3.1. Class discrimination with brain graphs

Class discrimination based on brain connectivity has gained an increasing interest in the last few years. Many of the studies in this direction, specially when using functional connectivity, are based on seed-region methods (Richiardi et al., 2013). This means that a seed voxel or region is defined and its correlation with other brain regions is analyzed. Despite this approach can be effective in specific applications, it is only sensitive to local changes in the brain connectivity networks and the selection of the seeds is strongly problem specific.

On the other hand, graph theory provides a powerful means to study the topological organization of the central nervous system, and is attracting increasing attention as a general and powerful framework to analyze brain connectivity networks (Bullmore and Sporns, 2009). According to Richiardi and Ng (2013) recent methods for discriminating brain graphs can be grouped into three overlapping categories: *network science, statistical hypothesis testing*, and *machine learning* approaches. The network science approach (Ekman et al., 2012; Ambrosen et al., 2013) looks for discriminative information from topological properties of the graphs, like node degree distribution, cluster coefficient, centrality indexes, among others (Brandes and Erlebach, 2005). The hypothesis testing approach (Zalesky et al., 2010; Ginestet et al., 2014; Kim and Pan, 2015) provides test statistics that are applicable to graphs or graph components like nodes and edges. A recent comparison of different statistical tests for group differences in functional connectivity was presented in Kim et al. (2014). Finally, the machine learning approach is based on the application of classifiers to the brain graphs. This is frequently complemented with hypothesis testing on the ability of the classifier to accurately discriminate between classes. Different classifiers have been used to discriminate between different diseases like Alzheimer (Wang et al., 2006; Chen et al., 2011), Depression (Craddock et al., 2009), and Schizophrenia (Shen et al., 2010). Moreover, in cognitive studies, different brain states or stimuli have been decoded by using classifiers (Richiardi et al., 2011; Tagliazucchi et al., 2012; Vega-Pons and Avesani, 2013; Vega-Pons et al., 2014). Given the increasing adoption of machine learning in the neuroimaging community (Richiardi et al., 2013), in this paper, we use this approach as the baseline for our proposals.

In the context of our particular problem, the class discrimination on *D*_*S*_ and *D*_*F*_ can be addressed by solving a binary graph classification problem. Despite graphs are flexible and rich data structures, they are difficult to handle. Therefore, the classification of graphs normally requires an intermediate step in which graphs are mapped into a vector space. The mapping can be done either implicitly by using graph kernels (Vishwanathan et al., 2010; Shervashidze et al., 2011) or explicitly by using the so-called embedding techniques (Riesen and Bunke, 2010; Gibert et al., 2012).

The selection of the most appropriate technique should be based on the intrinsic properties of the graph data at hand. In our case, we have graphs holding the FCVS property. Therefore, we are dealing with a particular and simplified case of the more general graph classification problem, in which a one-to-one node correspondence is already established. It is expected that tools like general purpose graph kernels or embeddings techniques are not optimal in this scenario, since they lack the ability of taking advantage of the FCVS property (Richiardi et al., 2013).

The most common approach for this kind of graph data is called *direct connection embedding* (DCE) (Richiardi et al., 2010). This is a simple embedding method in which a vector representation of a graph is obtained by unfolding the upper triangular part of its adjacency matrix. In other words, the DCE is a function *f* that takes a graph *G* with *l* nodes and adjacency matrix *A*, and maps it into a vector V_G_ = [*A_12_*,…, *A_ij_*] ∈ 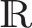
^*t*^ with *i* < *j* and $t=\frac{l\left(l-1\right)}{2}$.

After graphs are embedded into a vector space, the graph classification problem is transformed into a standard classification problem with vectorial data, where traditional machine learning classifiers can be directly applied.

Moreover, we can do hypothesis testing on the classification results, for both the structural and functional connectivity data. The null hypothesis *H*_0_ says that the classifier predicts at chance level and the binomial test can be used to test this hypothesis (Pereira et al., 2009). The binomial test is used under the assumption that the probability of a binary classifier predicting at random is *p* = 1/2. After a *k*-fold cross-validation is performed, a prediction for each sample in the dataset is obtained. Let $\hat{Y}=\left\{\hat{y_{1}},\ldots ,\hat{y_{n}}\right\}$ be the classifier predictions and *Y* = {*y*_1_, …, *y*_*n*_} the true labels. We can use as test statistic the number of misclassified samples $r=\overset{n}{\underset{i=1}{\sum }}I\left(y_{i},\hat{y_{i}}\right)$, where *I* is the indicator function that is equal to 1 if the prediction is equal to the true label and 0 otherwise. Then, the *p*-value can be computed by $Pr(i\leq r|H_{0})=\sum_{i=0}^{r}\left(\begin{array}{c} n\\ i \end{array}\right)p^{i}{\left(1-p\right)}^{n-i}$.

### 3.2. Kernel two-sample test for class discrimination

The classification based approach has at least two possible limitations. The first one lies on the need of splitting the data intro train/test sets. This could be problematic on small datasets (on the number of samples), as it is commonly the case on neuroimaging studies and specifically the case of the BTBR vs. B6 data we are studying. The second limitation comes from the nature of hypothesis testing on a *k*-fold cross validation procedure (Bengio and Grandvalet, 2004). Classifiers on different folds are trained on partially overlapped data and the test data in one-fold is used for training in another fold. Therefore, the i.i.d assumption on the samples in the binomial test is hardly satisfied.

Another way to look at the discrimination problem is through the two-sample test perspective. Two-sample tests have mainly been used in the low-dimensional context. However, the recently proposed *kernel two-sample test* (Gretton et al., 2012) provides a solution for high dimensional data or even data not defined in vector spaces, like graphs.

Given two random variables *X*_*A*_ and *X*_*B*_ with probability distributions *p*_*A*_ and *p*_*B*_, respectively, the KTST addresses the problem of determining whether the null hypothesis *H*_0_, saying that *p*_*A*_ = *p*_*B*_, is true or not, based on two samples $A=\left\{x_{1}^{A},\ldots ,x_{n}^{A}\right\}$ and $B=\left\{x_{1}^{B},\ldots ,x_{m}^{B}\right\}$ drawn from *p*_*A*_ and *p*_*B*_, respectively.

This test uses as test statistic the *Maximum Mean Discrepancy* (MMD) (Gretton et al., 2012), which in a general setting, is defined as

(1) $$MMD[F,p_{A},p_{B}]=\underset{f\in F}{\sup}(E_{X_{A}\sim p_{A}}[f(X_{A})]-E_{X_{B}\sim p_{B}}[f(X_{B})])$$

where F is a family of bounded continuous functions.

The quality of the MMD as a test statistic depends on the selection of the family of functions F. A convenient option is the unit ball in a characteristic reproducing kernel Hilbert space (RKHS) (Gretton et al., 2012). A RKHS is a Hilbert Space associated to a positive definite kernel function.

Given a non-empty set X, a *positive definite kernel*^1^ k:X×X→ℝ is a function that satisfies the *symmetry* and *positive definiteness* properties (Hofmann et al., 2008). It is known that if *k* is a kernel function, there is a mapping ϕ:X→H from X to some Hilbert space H, such that $k\left(x,x^{\prime }\right)={\langle \phi \left(x\right),\phi \left(x^{\prime }\right)\rangle }_{H}$ for all $x,x^{\prime }\in X$, where ${\langle \cdot ,\cdot \rangle }_{H}$ denotes the dot product in H. In this case, H is the RKHS associated to the kernel *k*. The notion of characteristic kernel was recently introduced in Fukumizu et al. (2009). It is a further restriction to the kernel function that guarantees that the MMD is a metric. This means that $MMD\left[F,p_{A},p_{B}\right]=0$ if and only if *p*_*A*_ = *p*_*B*_.

Therefore, studying whether the two distributions are different or not is the same as analyzing whether the associated MMD is equal to zero. In Sriperumbudur et al. (2011), it was proven that many popular kernels are characteristic, e.g., the Gaussian and Laplace kernels on 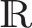
^*d*^ and therefore they are valid kernels for the KTST approach.

In practice, an unbiased estimate of *MMD*^2^ (Gretton et al., 2012) based on the observations *A* and *B* is:

(2) $$\begin{array}{l} MMD_{u}^{2}=\frac{1}{m(m-1)}\sum_{i\neq j}k(x_{i}^{A},x_{j}^{A})-\frac{2}{mn}\sum_{i,j}k(x_{i}^{A},x_{j}^{B})\\ \;+\frac{1}{n(n-1)}\sum_{i\neq j}k(x_{i}^{B},x_{j}^{B}) \end{array}$$

Different approaches were proposed in Gretton et al. (2012) to estimate the probability distribution of $MMD_{u}^{2}$ under the null hypothesis *H*_0_. In this paper, we follow the Monte Carlo approximation of the permutation test proposed in Olivetti et al. (2013). This is an iterative process, where in each iteration *i* = 1, …, *T*, elements from *A* and *B* are randomly exchanged to obtain *A*_*i*_, *B*_*i*_, and also the corresponding $MMD_{u,i}^{2}$ is computed. The sample $D_{0}=\left\{MMD_{u,1}^{2},\ldots ,MMD_{u,T}^{2}\right\}$ becomes an accurate approximation of the null distribution as the number of iterations *T* increases. Finally, the *p*-value is estimated as the fraction of elements in *D*_0_ equal or greater than the actual $MMD_{u}^{2}$ computed on the original observations *A* and *B*.

This test can be applied to any kind of data X, as long as a characteristic kernel is defined on X. In our problem, we are dealing with graph data, therefore we need a characteristic graph kernel. Next, we define a characteristic graph kernel for graphs with FCVS property based on the DCE method discussed in Section 3.1 and the Gaussian kernel.

Definition 3.1. Let G be a set of graphs containing *l* nodes and holding the FCVS property. Let *f*:G → 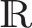
^*t*^, with $t=\frac{l\left(l-1\right)}{2}$ be the Direct Connection Embedding function that maps a graph into a real vector. The *direct embedding kernel* $k_{de}:G\times G$ → 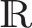 is defined as

(3) $$k_{de}(G_{1},G_{2})=\exp\left(-\frac{\|f(G_{1})-f(G_{2}){\|}_{2}^{2}}{2\sigma^{2}}\right)$$

Proposition 3.1. *The direct embedding kernel k_de_ is a characteristic positive definite kernel function*.

This proposition can be easily proven based on the fact that this kernel is the composition of the direct connection embedding (DCE) and the Gaussian kernel. The DCE is a bijective function for graphs with FCVS. This means that, working on the vector space obtained after the embedding is equivalent to working on the original graph space. Once the graph data is mapped into the vector space, the Gaussian kernel, which is a characteristic kernel, is applied. Therefore, the original *k*_*de*_ function is also a characteristic kernel.

Given the kernel function *k*_*de*_, we can apply the KTST directly on the graph data, either on the structural or functional connectivity. We will obtain an MMD, which is a measure of distance between samples in both classes, and the corresponding *p*-value.

An important characteristic of this approach is that it directly works on the whole dataset, i.e., splitting the data into train and test sets or a cross-validation procedure are not required. This property can be especially convenient in the case of small datasets, like the BTBR vs. B6 data we are studying.

### 3.3. Modality comparison

A quantitative comparison of the results of the application of classifiers or KTST on both structural and functional connectivity is not always straightforwardly possible. In the case of classifiers, the differences in accuracies can be misleading. For example, perfect classification results with both modalities would suggest that in both cases the classes are easily separable, but does not allow to determine whether there is still one of the two modalities for which the classes are more separated than the other. In the case of KTST, given the differences in nature between structural and functional connections, the MMD-values obtained from both modalities would have different null distributions, and therefore, their actual numerical values are not comparable.

One way of making the analysis of structural and functional connectivity comparable is by representing all connectivity data into a common space. Once all data is represented in the same space, test statistics like MMD would produce comparable results.

As we mentioned at the beginning of Section 3, the only difference between structural and functional connectivity data lies in the edge weighting function ω that measures the dependency between different brain regions.

Let Ω_*S*_ be the set of all weight values associated to all edges in the structural connectivity data, i.e., $\Omega_{S}={⋃}_{i=1}^{n}\left\{\omega_{i}^{s}\left(e\right)|e\in E_{i}^{s}\right\}$. In a similar way, we can define Ω_*F*_ as the set of all functional connectivity weight values $\Omega_{F}={⋃}_{j=1}^{m}\left\{\omega_{j}^{f}\left(e\right)|e\in E_{j}^{f}\right\}$. At this point, we assume that weight values in Ω_*S*_ are sampled i.i.d. from a continuous random variable *X*_*S*_ with an unknown distribution function and weights in Ω_*F*_ are i.i.d sampled from a continuous random variable *X*_*F*_ with also unknown distribution. This is a reasonable assumption since all weights of each modality have a common nature. As discussed in Section 2, structural weights are a measure of the number of white matter fibers connecting two brain regions. On the other hand, functional weights are the correlation between times series belonging to two brain regions.

Let *F*_*S*_(*x*) and *F*_*F*_(*x*) be the cumulative distribution functions of *X*_*S*_ and *X*_*F*_, respectively. It can be proven that both *F*_*S*_(*x*) and *F*_*F*_(*x*), if considered as new random variables, have uniform distribution in the interval [0, 1], i.e., *F*_*S*_(*x*) ~ *U*[0, 1] and *F*_*F*_(*x*) ~ *U*[0, 1]. This is actually true for any continuous cumulative distribution function (see Proposition 3.1 in Embrechts and Hofert, 2013). Therefore, values from *F*_*S*_(*x*) and *F*_*F*_(*x*) are directly comparable since they share the same distribution.

In practice, we compute the *empirical cumulative distribution function* (ECDF) of *X*_*S*_ as

(4) $$\hat{F_{S}}(x)=\frac{1}{\left|\Omega_{S}\right|}\sum_{\omega \in \Omega_{S}}I(\omega \leq x)$$

where *I*(ω ≤ *x*) is the indicator function, which is equal to 1 if ω ≤ *x* and 0 otherwise. Analogously, we compute the ECDF of *X*_*F*_ as

(5) $$\hat{F_{F}}(x)=\frac{1}{\left|\Omega_{F}\right|}\sum_{\omega \in \Omega_{F}}I(\omega \leq x)$$

Both ECDFs become accurate approximations of the true cumulative distribution functions as the cardinality of Ω_*S*_ and Ω_*F*_ increase. Notice that it is possible to obtain good approximations, even for datasets with small number of subjects, since $|\Omega_{S}|=O\left(l^{2}\cdot n\right)$ and $|\Omega_{F}|=O\left(l^{2}\cdot m\right)$, where *l* is the number of brain regions. For example, the BTBR vs. B6 dataset described in Section 2, which is composed of only 36 graphs, contains more than 30000 edges with non-zero weights.

Now, for each structural connectivity graph *G*^*s*^ = (*V*^*s*^, *E*^*s*^, ℓ^*s*^, ω^*s*^) we can compute a new graph $\hat{G^{s}}=\left(V^{s},E^{s},\ell^{s},\hat{\omega^{s}}\right)$, where nodes, edges, and the node labeling function remain the same and the only change is the edge weighting function. The new edge weighting function is defined as $\hat{\omega^{s}}\left(e_{s}\right)=\hat{F_{S}}\left(\omega^{s}\left(e_{s}\right)\right)$ for all $e_{s}\in E^{s}$. In an analogous way, for each functional connectivity graph *G*^*f*^ = (*V*^*f*^, *E*^*f*^, ℓ^*f*^, ω^*f*^), we compute the corresponding $\hat{G^{f}}=\left(V^{f},E^{f},\ell^{f},\hat{\omega^{f}}\right)$, where $\hat{\omega^{f}}\left(e_{f}\right)=\hat{F_{F}}\left(\omega^{f}\left(e_{f}\right)\right)$ for all $e_{f}\in E^{f}$. In this new graph representation, all edge weights are directly comparable since they share a common distribution. This means that we have mapped all graphs into a common representation space.

Given the new graph representation, we obtain a new structural dataset $\hat{D_{S}}=\left\{\hat{G^{s}},Y^{s}\right\}$ and a new functional dataset $\hat{D_{F}}=\left\{\hat{G^{f}},Y^{f}\right\}$. We define $MMD_{S}^{2}$ and $MMD_{F}^{2}$ as the $MMD_{u}^{2}$ computed on $\hat{D_{S}}$ and $\hat{D_{F}}$, respectively^2^. Furthermore, we can compute a unique null distribution taking into account all graphs $\left(\hat{G^{s}}\cup \hat{G^{f}}\right)$ since they all are in a common space. At this point, we can directly compare the $MMD_{S}^{2}$ and $MMD_{F}^{2}$ quantities since they share a unique null distribution.

Moreover, we can define a new test statistic that directly addresses the question of whether the structural data is more discriminative than the functional data. This test statistic is $MMD_{SF}^{2}=MMD_{S}^{2}-MMD_{F}^{2}$. We can also use the Monte Carlo approximation of the permutation test described in Section 3.2 for the estimation of the null distribution of $MMD_{SF}^{2}$. First of all, we split the data into the following four groups:

- $D_{S}^{A}$: Structural data belonging to the first class (BTBR).
- $D_{F}^{A}$: Functional data belonging to the first class (BTBR).
- $D_{S}^{B}$: Structural data belonging to the second class (B6).
- $D_{F}^{B}$: Functional data belonging to the second class (B6).

At each iteration *i* = 1, …, *T* we randomly exchange data from all groups to obtain $D_{S,i}^{A}$, $D_{F,i}^{A}$, $D_{S,i}^{B}$, and $D_{F,i}^{B}$. Then, we compute $MMD_{S,i}^{2}$ from $D_{S,i}^{A}$ and $D_{S,i}^{B}$, and $MMD_{F,i}^{2}$ from $D_{F,i}^{A}$ and $D_{F,i}^{B}$ in order to obtain $MMD_{SF,i}^{2}=MMD_{S,i}^{2}-MMD_{F,i}^{2}$. Finally, the *p*-value can be estimated as the fraction of elements in $\left\{MMD_{SF,1}^{2},\ldots ,MMD_{SF,T}^{2}\right\}$ that are equal or greater than the $MMD_{SF}^{2}$-value computed on the original (unpermuted) data.

## 4. Results

**Figure 1:**
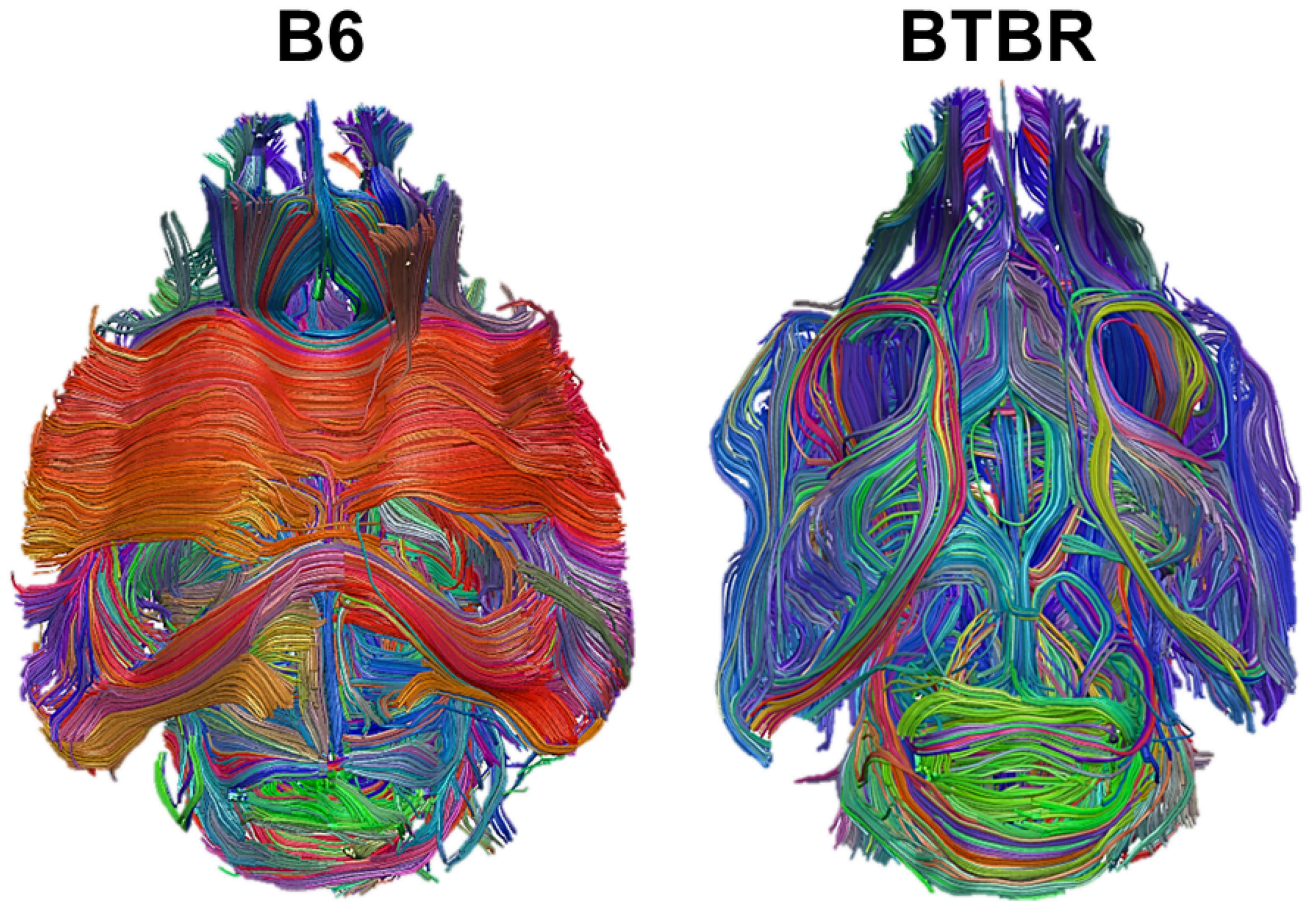
**Major white matter reorganization in BTBR mice**. Diffusion tensor tractography of white matter in a representative B6 (left) and BTBR (right) subject. The large white matter bundles (in red in the left panel) denote the Corpus Callosum and the posterior Hippocampal Commissure, which are absent in the BTBR (right panel).

In this section, we first start with a visual inspection of the BTBR vs. B6 dataset. In Figure 1, we report the Diffusion Tensor Tractography data for B6 and BTBR mice. This representation highlights the overall rearrangement of white matter in BTBR mice, including the lack of inter-hemispheric connections in the corpus callosum and dorsal hippocampal commissure, together with a rostro-caudal reorganization of white matter tracts in these animals.

**Figure 2:**
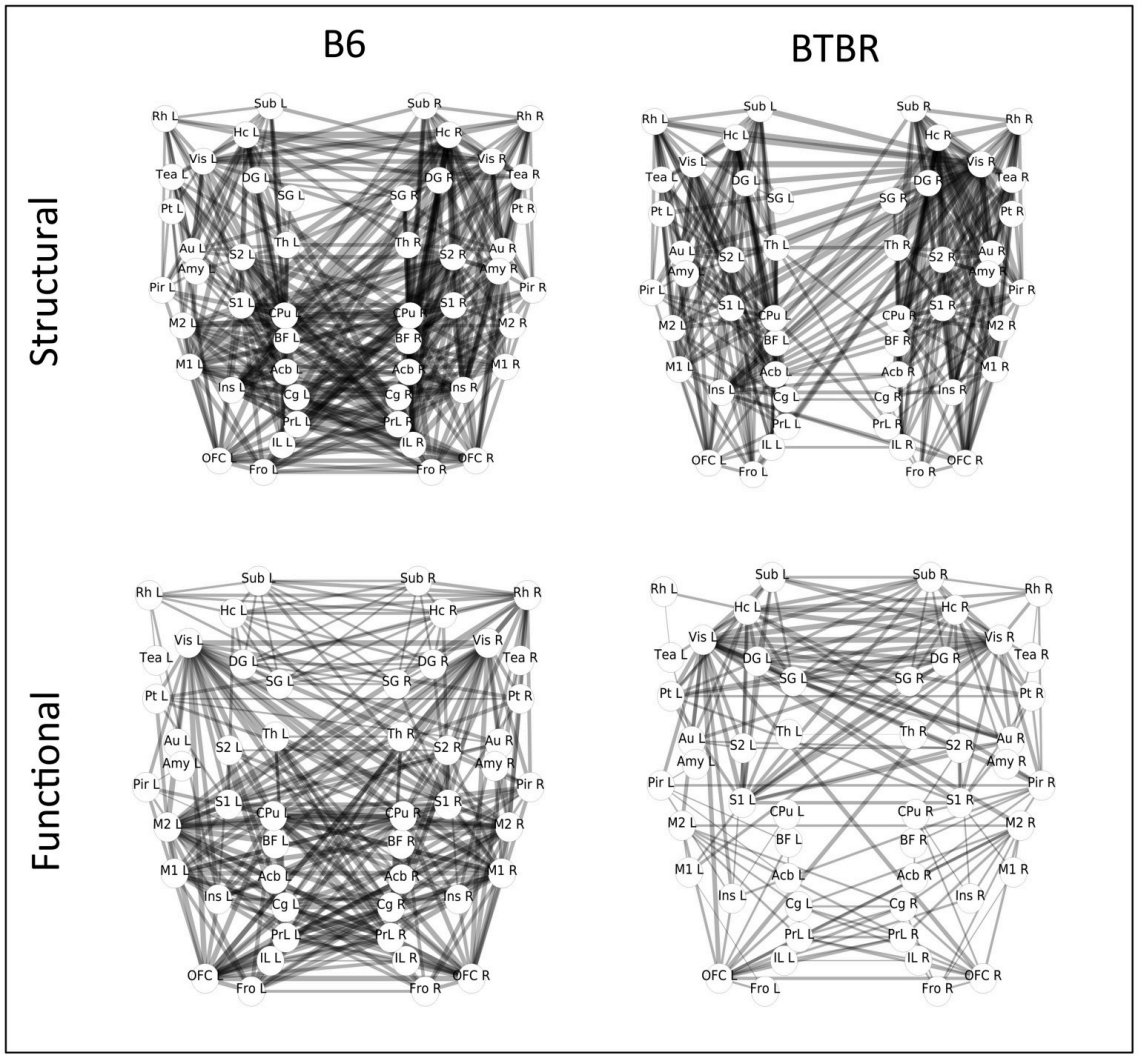
**Graph representation of group-level structural and functional connectivity in the BTBR mouse line, and in the control B6 line**. The labels indicate the brain regions corresponding to the nodes of the network, and the weights of the connecting lines indicate the strength of the pairwise connections. The graph represents a top view of the mouse brain, with the anterior part of the brain pointing down, and the two hemispheres on the left and right side, respectively.

Moreover, group-level structural connectivity matrices for the BTBR and B6 groups were calculated averaging the individual subjects matrix elements. In the case of functional connectivity, subject-wise adjacency matrices were Fisher's transformed, averaged across subjects and back-transformed to create group-average average correlation matrices for the BTBR and B6 groups. Both the resulting average structural connectivity graphs and the average functional connectivity graphs are shown^3^ in Figure 2.

From Figure 2 we observe that, in the case of structural connectivity (top panel), the reduction in number and strength of interhemispheric connections in the BTBR line (left panel) is accompanied by a reorganization of longitudinal tracts. On the other hand, functional connectivity network in the BTBR (botton left panel) is sparser in terms of number of edges, the distribution and strength of connections is remodulated, and the network shows an overall symmetrical pattern of interhemispheric homotopic connectivity. This figure suggests that the structural connectivity is more affected than the functional connectivity in the BTBR class. Next, we use the analysis methods introduced in Section 3 to analyze this dataset.

We first address the class discrimination problem using structural and functional connectivity independently. Then, we address how informative the two modalities (structural and functional) are by computing the common space representation and applying the proposed $MMD_{SF}^{2}$ test statistic.

In order to apply the proposed methods, we need edge weights to be a similarity measure, i.e., the higher the weight values the stronger the dependency between nodes. Structural connectivity weights hold this property, but raw correlation values in functional connectivity does not, and therefore should be preprocessed. We have follow different preprocessing approaches like thresholding correlations with a value in the range [0, 0.5] or using the absolute value of negative correlations. In all cases, we have obtained the same general conclusion in terms of interpretation of *p*-values, even though numerical results have been slightly different. For simplicity, we are presenting here the experiments where only possitive correlations were kept, i.e., using a threshold equal to zero.

In all calculations, we use the graph kernel *k*_*de*_ introduced in Section 3.2 as a similarity measure between graphs. This graph kernel allowed us to use kernel based classifiers like Support Vector Machines (SVM) and also the Kernel Two-Sample Test (KTST), directly on the graph data. The parameter σ of this kernel was set to the median value of the distances between the vectors resulting from the direct connection embedding (DCE) of the graphs. The use of the mean distance is a standard heuristic in the case of low sample size, since no extra labeled data is needed to estimate this parameter.

For all classification experiments, we used SVM classifier within a leave-one-subject-out cross-validation approach, i.e., in each fold, we trained the classifier with all but one subject and tested on the data of that remaining subject. We report the mean classification accuracy across folds and also the *p*-value computed by using the binomial test as explained in Section 3.1. In the case of the KTST experiments, we used *T* = 100000 as the number of iterations in the computation of the null distribution. We report the $MMD_{u}^{2}$ distance and the corresponding *p*-value.

All the code used in the experiments, which generates the results reported in the tables and figures in this section, was developed in Python using the numerical libraries NumPy and SciPy, together with the machine learning package Scikit-learn. Our code is available under a Free / OpenSource license^4^.

### 4.1. Class discrimination on single modalities

The results of the application of the leave-one-subject-out cross-validation and the KTST on structural *D*_*S*_ and functional *D*_*F*_ datasets are presented in Table 1.

**Table 1:** **Leave-one-subject-out cross-validation (classification approach) and KTST results on the structural and functional connectivity datasets**.

	**Classification**		**KTST**	
**Modality**	**Accuracy (std)**	***p*****-value**	**$MMD_{u}^{2}$**	***p*****-value**
Structural	1.0 (0.0)	7.6 × 10^−6^	0.64	1.0 × 10^−5^
Functional	0.9 (0.2)	2.0 × 10^−5^	0.12	1.0 × 10^−5^

**Figure 3:**
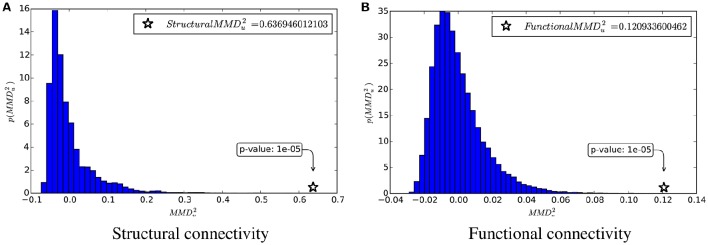
**$MMD_{u}^{2}$ and the estimation of its null distribution. (A)** Structural connectivity. **(B)** Functional connectivity.

Moreover, the null distribution and $MMD_{u}^{2}$-values computed on the structural and functional data are shown in Figure 3.

**Figure 4:**
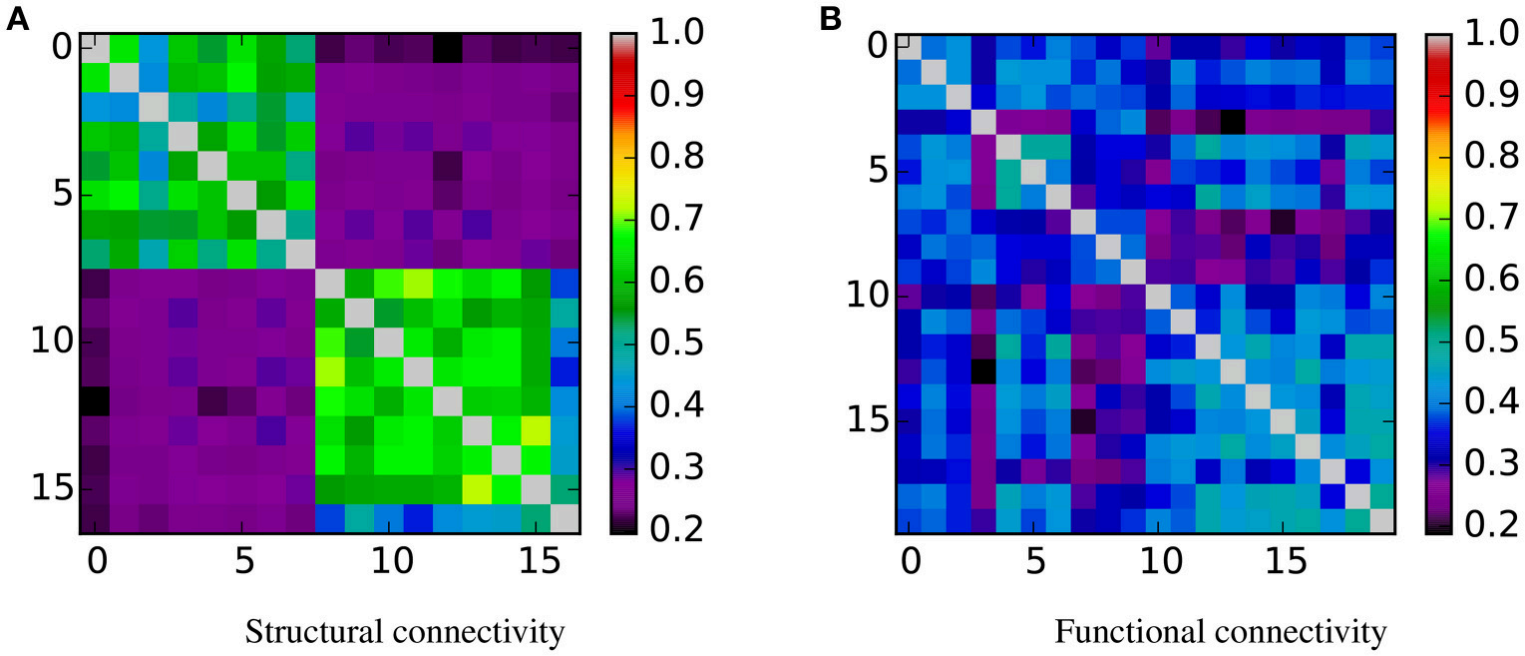
**Similarity matrices**. Rows and columns are organized by class, first the BTBR samples, and then the B6. In the case of structural connectivity, it is clear that samples from the same class are similar between them and dissimilar to the samples from the other class. For the functional connectivity this pattern is not evident. **(A)** Structural connectivity. **(B)** Functional connectivity.

From these table and figure we note that there is a perfect agreement between the two approaches. With both SVM and KTST it is possible to discriminate between the BTBR and B6 classes. In both cases the results are very significant, which means that the two classes are easily separable by independently using the structural and the functional connectivity. However, at this point it is still not clear which connectivity data is more separable and if the potential difference is significant or not. Inspection of the kernel matrices in Figure 4 shows a clear pattern of class separation in the structural kernel matrix, while such separation pattern is much less evident in the functional kernel matrix. This suggests that structural connectivity data should be more discriminative than the functional one. However, the application of standard methods like SVM or the KTST does not provide a reliable way of quantifying this potential difference.

### 4.2. Modality comparison

In order to test how discriminative the two modalities are, we have mapped all connectivity data in the common representation space as described in the Methods Section, and computed the structural $MMD_{S}^{2}$ and functional $MMD_{F}^{2}$-values. These values share a common null distribution and therefore are directly comparable. Moreover, we have computed the $MMD_{SF}^{2}$ that directly measures the differences between the two types of connectivity. These results are reported in Table 2.

**Table 2:** **Application of KTST on the common representation space of connectivity data**.

**Structural**		**Functional**		**Difference**	
**$MMD_{S}^{2}$**	***p*****-value**	**$MMD_{F}^{2}$**	***p*****-value**	**$MMD_{SF}^{2}$**	***p*****-value**
0.43	1.0 × 10^−5^	0.11	0.00355	0.32	0.00074

*Computation of $MMD_{S}^{2}$, $MMD_{F}^{2}$, and $MMD_{SF}^{2}$ with the corresponding p−values*.

**Figure 5:**
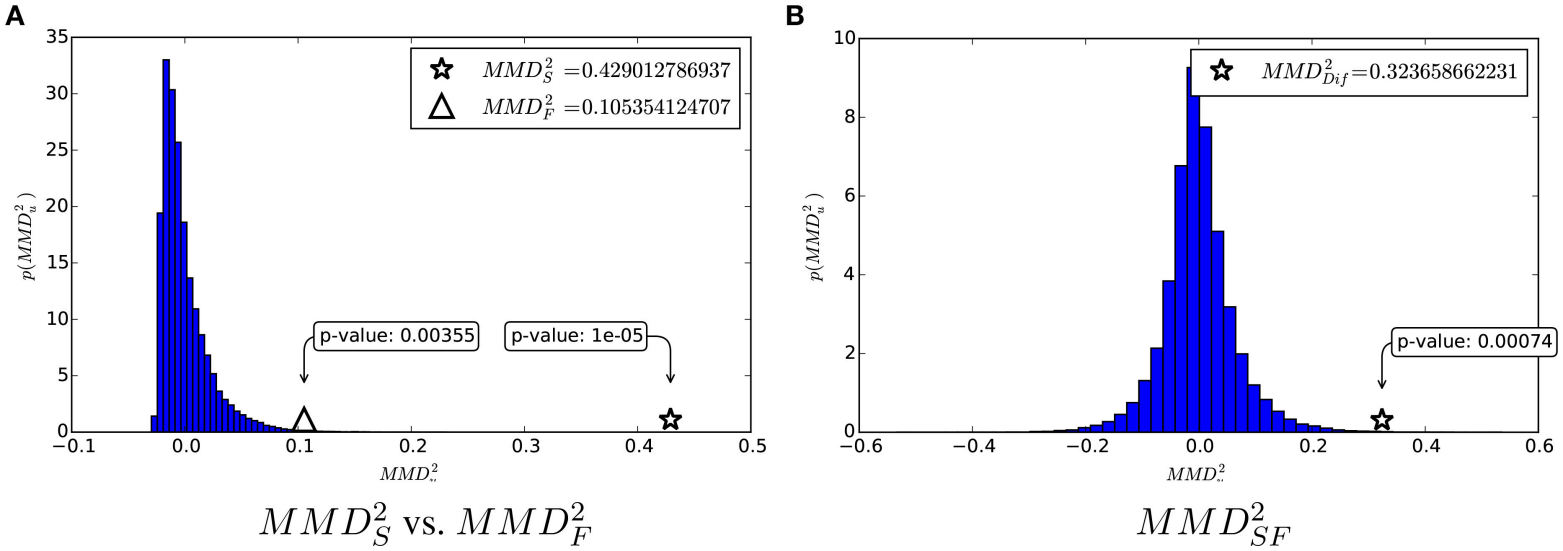
**(A)** Comparison of structural $MMD_{S}^{2}$ and functional $MMD_{F}^{2}$ according to their common null distribution. **(B)** $MMD_{SF}^{2}$ and its null distribution.

In Figure 5, we first show the $MMD_{S}^{2}$ and $MMD_{F}^{2}$ within their null distribution, for a visual comparison of the two quantities. Moreover, we show the proposed test statistic $MMD_{SF}^{2}$ and its estimated null distribution.

These analyses corroborate the idea that both structural and functional connectivity enable discrimination of the two classes, as the *p*-values associated to $MMD_{S}^{2}$ and $MMD_{F}^{2}$ are both below standard thresholds for significance (0.05 and 0.01). However, the fact that $MMD_{S}^{2}>MMD_{F}^{2}$ means that the structural connectivity is more informative than the functional connectivity for the class discrimination problem. Such a difference is quantified by $MMD_{SF}^{2}$ and its corresponding *p* = 0.00069 tells that it is very significant.

## 5. Discussion

The independent application of the KTST and classifiers to both connectivity modalities (Section 4.1) shows that both structural and functional connectivity are very informative for the class discrimination problem. This result corroborates previous findings with this dataset (Dodero et al., 2013; Sforazzini et al., 2016). More specifically, in Dodero et al. (2013) the structural connectivity was characterized, showing large alterations in white-matter organization, including lack of courpus callosum and hippocampal commissure, with a degree of interhemisphreic connectivity maintained by the anterior commissure. Moreover, a reduction in intra-hemispheric fronto-cortical functional connectivity was reported in Sforazzini et al. (2016), although the functional inter-hemispheric connectivity was preserved in the posterior sensory cortical areas.

Those previous studies with this dataset also suggested the idea that the functional connectivity was less affected than the structural connectivity in the BTBR model. However, there was no way to quantify this difference. The results of the class discrimination problem obtained in Section 4.1 are not a solution to this modality comparison problem. The fact that both modalities produce very significant results in class discrimination does not provide evidence about which modality is more informative and therefore more affected.

The common representation space and the test statistic presented in this paper directly address this modality comparison problem. Their use, described in Section 4.2, allowed us to quantify the difference between modalities and we found that such difference is very significant. We can state that this is the key result of this study, and implies that the difference between the BTBR line and its B6 background line is larger for structural connectivity than for functional connectivity. This is consistent with the qualitative observation that homotopy is partially preserved in the functional connectivity patterns exhibited by the BTBR mouse line, in spite of the lack of Corpus Callosum.

This evidence supports the idea that the organization of functional connectivity networks has a very robust topology that can tolerate substantial alterations in the underlying structural connectivity substrate. Indeed, a number of studies in humans and animal models have shown that functional connectivity networks present hierarchically modular structures and a scale free topology (Bullmore and Sporns, 2009). It has been suggested that this small-world organization provides the brain with an efficient network of information trafficking, whose connectedness is preserved also in the presence of certain degree of miswiring (Achard and Bullmore, 2007). The BTBR mouse line provides a striking example of this phenomenon.

The results obtained in this paper may have important implications for our understanding of the interplay between functional and structural connectivity. While studies in healthy subjects support the view that structural connectivity, to a large extent, constrains and predicts functional connectivity, a model like the BTBR, where white matter is dramatically reorganized, seems to challenge this notion. This support previous studies like (O'Reilly et al., 2013), where the surgical rescission of the corpus callosum in monkeys was shown to produce an acute, post-operative impairment of functional connectivity that can be recovered in the months after surgery, possibly due to a reorganization of functional connections driven by plastic processes. Similarly, developmental neuroimaging studies have shown that a direct correlation between these two measures of connectivity does not hold in children, as a structure-function relationship appears to mature with age (Supekar et al., 2010). In general, the organization of functional connectivity appears to be relatively robust to changes in white matter structure as dramatic as the lack of Corpus Callosum.

While the mechanisms whereby homotopic organization of functional connectivity appears to be preserved in the BTBR mouse are not precisely known, a few hypotheses can be put forward. The anterior and posterior commissures, two white matter bundles that are preserved in the BTBR model, may compensate, at least partially, for the lack of Corpus Callosum. Moreover, ascending brainstem or thalamic projections may contribute to maintaining interhemisphere connectivity in the acallosal mice. Finally, very weak long distance structural links, as demonstrated by recent anatomical tract tracing studies, see Oh et al. (2014), may support long distance interactions that favour integration of functional conenctivity even in the absence of major white matter tracts.

Clearly, this is very preliminary evidence that requires a more extensive experimental validation and needs to be reproduced in human studies before strong conclusions can be drawn. Moreover, alternative interpretations of the results cannot be completely discarded. For example, strong differences in signal to noise ratio could also explain the observed differences in discrimination power between the two connectivity modalities. We consider that the effect of noise in the networks should be carefully studied and will be part of our future perspectives. An additional factor that may affect the inference on brain connectivity networks is the threshold to filter out non-relevant edges in the networks. We didn't investigate how different choices of threshold values are related to the subsequent inference. Such investigation would require to consider several methods of graph encoding, a question beyond the scope of this work. We also note that functional connectivity was defined on the basis of Pearson coefficients. It has been pointed out (Friston, 2011) that this widely used definition includes pairwise correlations that may be driven by third party input, and that other measures of correlation or effective connectivity may provide more accurate estimates of indirect influences. Application of the method hereby proposed to other measures of correlation may be helpful to elucidate the origin of functional connections between structurally unconnected regions.

Despite the data analyzed in this study have not been collected pairwised in a within-subject design, the inference on multimodal brain connectivity may take advantage of the correspondence between structural and functional network. It is worthwhile to remark that the current formulation of the Two Kernel Sample Test can not manage such property of the data. Finally, we note that the structural connectivity dataset used for this analysis relies on FACT deterministic algorithm for white matter tractography. Recent advances in tractography with diffusion MRI have been proposed (see, e.g., Soares et al., 2013, Daducci et al., 2016) that may enable refinement of the structural connectivity network and improved resolution of crossing fibers. However, the broad connectional differences observed by Dodero et al. (2013) and their investigations appear to be more than adequate to capture the dramatic structural differences between the BTBR mouse and its control. In keeping with this, more advanced mapping methods—i.e., HARD at 16.7 T—have recently revealed structural changes largely overlapping with the findings reported in Fenlon et al. (2015).

Besides structural and functional connectivity, a number of different brain connectivity networks have also been defined, e.g., effective connectivity, co-activation, and metabolic co-variance networks. All of these measures of connectivity capture different aspects of the complex structure of interconnections between the anatomical and functional elements. The methods proposed in this paper are not restricted to the particular case of structural and functional connectivity comparison and nor to the specific BTBR model data. In a more general sense, these methods can be applied to any kind of graph data holding the fixed cardinality vertex sequence (FCVS) property. Therefore, our approach provides a general method to assess the differential effects of disease states, neuropsychiatric conditions, genetic background etc. on diverse brain networks. Thus, enabling the use of connectivity measures as markers of disease or response to treatment, and the study of the interrelations between different types of connectivity.

## 6. Conclusions

In this paper, we have proposed the use of the kernel two-sample test (KTST) for the class discrimination problem based on brain connectivity data. We have shown that, given a characteristic graph kernel, the KTST can be directly applied to assess whether two populations of brain graphs belong to the same class or not. This method was applied to (BTBR vs. B6) mice datasets by using both structural and functional connectivity graphs. We found that for both connectivity modalities, the differences between classes were very significant.

Moreover, we have studied the dependency between alterations in the structural and functional connectivity. We have shown that the results of the class discrimination problem based on single modalities are not straightforwardly comparable. Therefore, we introduced a common representation space for structural and functional connectivity, that makes KTST results on both modalities directly comparable. Additionally, we have defined a new test statistic to quantify the difference between the two modalities. The application of this test statistic showed that the structural connectivity is significantly more affected than the functional connectivity in the BTBR model. This finding supports the idea that functional connectivity networks are tolerant with respect to alterations of the underlying structural connectivity.

Even though the main goal of this work was to study the interrelation between structural and functional connectivity in the BTBR mice model, the methods proposed here are not restricted to this particular case study. As future works, we plan to extend this analysis to human data in order to collect more evidence about the interrelation between the two modalities. Moreover, the proposed methods can be directly applied to more general kinds of graphs, particularly, from other types of brain connectivity. Therefore, our method provides a general framework to assess and compare the effects of brain conditions and diseases on differently defined forms of connectivity, including structural, functional, and anatomical.

## Author contributions

SV, EO, PA, and AB designed the analysis methods and conceived the experiments. SV conducted the experiments. LD and AG collected and pre-processed the data. SV and AB wrote the paper. All authors reviewed the manuscript.

### Conflict of interest statement

The authors declare that the research was conducted in the absence of any commercial or financial relationships that could be construed as a potential conflict of interest.

## Footnotes

^1^Hereafter called *kernel* for simplicity.

^2^$MMD_{S}^{2}$ and $MMD_{F}^{2}$ are still unbiased estimations of *MMD*^2^, the *u* subindex is removed for the sake of simplicity in the notation.

^3^The description of the labels of the nodes in Figure 2 is given as Supplementary Material.

^4^https://github.com/svegapons/ktst_struct_func
