# Supplementary material for: Differential Effects of Brain Disorders on Structural and Functional Connectivity
Source: Front Neurosci. 2017 Jan 9;10:605. doi: 10.3389/fnins.2016.00605 (PMC5221415; doi:10.3389/fnins.2016.00605)
Supplement: Supplementary file 1 [file Presentation1.pdf]

Supplementary Material:

Differential Effects of Brain Disorders on Structural and Functional Connectivity

Sandro Vega-Pons*, Emanuele Olivetti, Paolo Avesani, Luca Dodero, Alessandro Gozzi and Angelo Bifone

*Correspondence:
Author Name: Sandro Vega-Pons
sv.pons@gmail.com

1 DESCRIPTION OF NODE LABELS IN FIG. 1 AND 2

Acb R: accumbens nucleus dx;

Acb L: accumbens nucleus sx;

Amy R: amygdala dx;

Amy L: amygdala sx;

Au R: auditory cortex dx;

Au L: auditory cortex sx;

BF R: basal forebrain dx;

BF L: basal forebrain sx;

Cg R: cingulate cortex dx;

Cg L: cingulate cortex sx;

Cpu R: caudate putamen dx;

Cpu L: caudate putamen sx;

DG R: dentate gyrus dx;

DG L: dentate gyrus sx;

Fro R: frontal association cortex dx;

Fro L: frontal association cortex sx;

HC R: posterio-ventral hippocampus dx;

HC L: posterio-ventral hippocampus sx;

IL R: infralimbic prefrontal cortex dx;

IL L: infralimbic prefrontal cortex sx;

Ins R: insular cortex dx;

Ins L: insular cortex sx;

M1 R: motor cortex 1 dx;

M1 L: motor cortex 1 sx;

M2 R: motor cortex 2 dx;

M2 L: motor cortex 2 sx;

OFC R: orbitofrontal cortex dx;

OFC L: orbitofrontal cortex sx;

PrL R: prelimbic prefrontal cortex dx;

PrL L: prelimbic prefrontal cortex sx;

Pt R: parietal association cortex dx;

Pt L: parietal association cortex sx;

Pir R: piriform cortex dx;

Pir L: piriform cortex sx;

Rh R: rhinal cortex dx;

Rh L: rhinal cortex sx;

S1 R: primary somatosensory cortex dx;

S1 L: primary somatosensory cortex sx;

S2 R: secondary somatosensory cortex dx;

S2 L: secondary somatosensory cortex sx;

SG R: stratus granulosum dx;

SG L: stratus granulosum sx;

Sub R: subiculum dx;

Sub L: subiculum sx;

TeA R: temporal association cortex dx;

TeA L: temporal association cortex sx;

Th R: thalamus dx;

Th L: thalamus sx;

Vis R: visual cortex dx;

Vis L: visual cortex sx.
